# Bio-inspired aptamers decorated gold nanoparticles enable visualized detection of malathion

**DOI:** 10.3389/fbioe.2023.1165724

**Published:** 2023-03-03

**Authors:** Peng Li, Haonan Zhan, Sijian Tao, Zhuohao Xie, Jiahao Huang

**Affiliations:** ^1^ School of Biomedical Engineering, Southern Medical University, Guangzhou *,* China; ^2^ Department of Critical Care Medicine, Affiliated Hospital of Guangdong Medical University, Zhanjiang *,* China

**Keywords:** bio-inspired recognition components, super-hydrophilic nanomaterial, biosensor, enzyme-free system, colorimetric assay

## Abstract

Biosensors always respond to the targets of interest in a specific manner, employing biological or bio-mimic recognition elements such as antibodies and aptamers. Inspired by target recognition in nature, an aptamer-mediated, gold nanoparticle-based sensing approach is developed in this work for effective determination of malathion. The sensing system consists of negatively charged aptamer probes, and polycationic proteins, protamine, as well as exceptional colorimetric nanoprobes, barely gold nanoparticles (AuNPs). Protamine molecules bound to aptamer probes hinder the aggregation of AuNPs, while no such inhibition is maintained when aptamer-specific malathion is introduced into the solution, thus leading to the solution colour change from red to blue observable by the naked eye. The assay is accomplished *via* a mix-and-measure step within 40 min with a detection limit as low as 1.48 μg/L (3σ/s rule). The assay method also exhibits high selectivity and good applicability for the quantification of malathion in tap water with recovery rates of 98.9%–109.4%. Additionally, the good detection accuracy is also confirmed by the high-performance liquid chromatography method. Therefore, the non-enzymatic, label- and device-free characteristics make it a robust tool for malathion assay in agricultural, environmental, and medical fields.

## 1 Introduction

In living system, biological particles, such as cells and virus, always respond to their relevant receptors in a specific manner by employing target recognition elements such as antibodies and aptamers. Inspired by the target recognition in biological particles, an aptamer-decorated super-hydrophilic gold nanoparticle-based sensing approach is developed in this work for effective determination of malathion. Malathion, as one of the most frequently used and broad-spectrum organophosphate pesticides (OPs) (Vasseghian et al., 2022), has a great impact on improving the productivity of crops, including wheat, grain, rice, and peanuts, and so forth. However, many studies have demonstrated that malathion is strongly related to the occurrence of many diseases, such as Alzheimer’s disease (Venkatesan et al., 2017), systemic toxicity (Abdo et al., 2021), and cancer (Anjitha et al., 2020). The excessive use of OPs has brought about many serious problems and aroused great public concern in environmental protection, ecological balance, food safety, and human health. According to the Guidelines for Canadian Drinking Water Quality, the maximum acceptable concentration (MAC) of malathion in drinking water is set at 290 μg/L. And the government in China has also issued the standard of malathion in drinking water (GB 5749-2006) at 250 μg/L (Sang et al., 2022). It is thereby urgently demanded to develop effective methods for malathion quantification (Albuquerque and Poppi, 2015; Zhang et al., 2018; Wang et al., 2019).

A variety of conventional methods have been utilized for the determination of malathion at trace level, including gas chromatography (GC) (Bavcon et al., 2003), high-performance liquid chromatography (HPLC) (Bazmandegan-Shamili et al., 2017), gas chromatography-mass spectrometry (GC-MS) (Latifah et al., 2011), and so on. Nevertheless, they suffer from certain drawbacks, since these methods are highly dependent on the applications of costly and sophisticated equipment, which always need to be operated by skilled personnel in a relatively time-consuming fashion. Considerable effort has thus devoted to developing fast and accurate approaches for malathion analyses. Enzyme-linked immunosorbent assays (ELISA) (Brun et al., 2005; Tamarit-López et al., 2011) represent one of the most typical and powerful strategies among them. In ELISA, antibodies are indispensable for analyte recognition and subsequent signal reporting in ELISA for malathion detection. However, antibodies are difficult to obtain, highly expensive, and easily denatured. ELISA also involves many tedious washing and separation steps (Jaria et al., 2020). Furthermore, sensitive biosensors have been constructed for malathion assay *via* the utilization of acetylcholinesterase (AChE) (Raghu et al., 2014; He et al., 2018; Bao et al., 2019; Chen et al., 2021), which serves as important analyte recognition element due to the remarkable activity inhibition of AChE by malathion. However, the sensing mechanisms are always established in a turn-off manner, which easily causes undesired false results. Other than this, the AChE activity is easily affected by many unexpected factors, such as high temperature and harsh buffer solution, which may ruin the detection reliability. Therefore, enzyme-free strategies are superior in terms of assay cost, simplicity of experimental procedures, and detection reliability.

To circumvent the potential problems mentioned above, aptamer can provide an ideal solution. Aptamer is single-strand oligonucleotide probe achieved by *in vitro* selection process called systematic evolution of ligands by exponential enrichment (SELEX) (Meng et al., 2015), which targets to a wide range of analytes, such as small molecules (Lou et al., 2019; Luo et al., 2019), metal ions (Raducanu et al., 2020), proteins (Lo et al., 2021), tumor markers (Li et al., 2022), and even the whole cells (Lv et al., 2021). Aptamer possesses many advantages, such as ease of synthesis and modification, small size, high affinity, low cost, high thermal stability, wide types of targets, and compatible for many signal reporting techniques. The last decade has witnessed the great progress in the development of aptasensors for malathion monitoring, which are usually coupled with colorimetric (Bala et al., 2016; Bala et al., 2017), fluorescent (Bala et al., 2018; Huang et al., 2021), electrochemical (Xu et al., 2019; Xu et al., 2021b), and chemiluminescent (Wu et al., 2021) techniques. Colorimetric detection methods have attracted special attention due to the instrument-free, easy-to-use, and low-cost features (Guo et al., 2021; Wu et al., 2022). It is thus of great importance to develop simple yet robust methods for malathion analysis.

Toward this goal, we herein propose an efficient colorimetric aptasensor for malathion detection, which is an antibody- and enzyme-free sensing system and can be performed in a washing-and device-free fashion. There are three species in the sensing system, including aptamer probes, protamine molecules, and unmodified gold nanoparticles (AuNPs). Aptamer probes are used as specific ligands for malathion recognition, and AuNPs serving as extremely sensitive colorimetric indicators. Furthermore, protamine molecules play an essential role in modulating the interaction among AuNPs, aptamer probes, as well as malathion. The presence of malathion breaks the absorption balance between aptamer probes and protamine molecules, which subsequently induces aggregation of AuNPs followed by an obvious colour change ready for visual detection. The sensing of malathion is conducted in a mix-and-measure manner and response signals can be monitored by using the naked eye. The assay can be completed within 40 min with a detection limit of 1.48 μg/L, which is far below the standards of malathion in drinking water issued by the Chinese and Canadian governments. Therefore, we report a novel sensing method for malathion analysis, which is ease-to-use, inexpensive, and selective. The present work may find potential applications in agriculture, food safety, and human health.

## 2 Experimental section

### 2.1 Chemicals and materials

Chloroauric acid trihydrate (HAuCl_4_) and trisodium citrate were both purchased from Macklin Biochemical Co. (Shanghai, China). Protamine sulfate salt was purchased from Sigma-Aldrich Co. (St. Louis, MO, United States). Organophosphorus pesticides, including malathion, acetamiprid, carbaryl, chlorpyrifos, methamidophos, and imidacloprid, were all obtained from Alta Scientific Co. (Tianjin, China). The aptamer probes of malathion with the sequences of 5′-AGC TTG CTG CAG CGA TTC TTG ATC GCC ACA GAG CT-3′, were ordered from Sangon Biotechnology Co. (Shanghai, China). All the reagents used were of analytical grade and were used as obtained without further purification. All the solution was prepared by using ultrapure water (resistivity≥18.2 MΩ cm) supplied by a Thermo Fisher Scientific GenPure water purification system (United States). The glassware was rinsed with aqua regia prior to use.

UV-vis absorption spectra were recorded using a Thermo Fisher Scientific Evolution 300 spectrophotometer (United States). UV-vis absorption spectra were scanned from 400 nm to 800 nm and then collected for further analysis. Dynamic light scattering (DLS) data and Zeta potential were obtained using a Brookhaven Instruments NanoBrook 90PlusZeta (United States). The transmission electron microscope (TEM) analyses were conducted by utilizing Hitachi H7800 microscope (Japan).

### 2.2 Synthesis of AuNPs

AuNPs were synthesized following the trisodium citrate reduction method with slight modifications (Grabar et al., 1995; Elghanian et al., 1997). Briefly, an aqueous of 0.01% HAuCl_4_ (100 mL) solution was heated to boiling before 3 mL 1% trisodium citrate solution was quickly introduced under vigorous stirring (450 rpm). The mixture solution kept boiling for an additional 20 min. The solution colour gradually changed from pale yellow to light gray and eventually turned into wine red. This indicated the generation of AuNPs. The mixture was naturally cooled at room temperature with stirring and then stored in dark bottles at 4°C for further use. The UV-vis absorption spectra and Zeta potential were recorded to indicate the properties of AuNPs. DLS data and TEM image were used to determine the average size of the AuNPs.

### 2.3 Detection of malathion

The serial dilutions of malathion were prepared by introducing stock solution of malathion in acetone into phosphate buffer solution (pH 7.2) and then stored at −20°C before use. For malathion assay, malathion-specific aptamer (3 μL, 25 μM) was mixed with various concentrations of malathion, diluted with phosphate buffer, and then incubated for 10 min at room temperature. Next, protamine (3 μL, 50 μg/mL) was added and incubated for another 20 min. Finally, AuNPs (400 μL) were introduced into the above mixture and remained still for 10 min. Afterward, the absorption spectra were collected by using UV-vis spectrophotometer. The images of reaction samples were also taken for comparison. Moreover, the particle sizes were characterized and recorded. To study the assay selectivity, various un-specific organophosphorus pesticides such as acetamiprid, carbaryl, chlorpyrifos, methamidophos, and imidacloprid were also used as analogues to test the detection performance of the strategy.

### 2.4 Determination of malathion in spiked sample

Tap water directly taken from laboratory water outlet at Southern Medical University was chosen to evaluate the validity of the proposed strategy for real application. After filtered through a 0.45 μm membrane, the filtered tap water was spiked with malathion with concentrations of 10 μL and 50 μg/L to prepared the spiked samples. The as-prepared positive samples were then analysed according to the experimental procedure described above. And the theoretical values were calculated according to the established standard calibration curve.

## 3 Results and discussion

### 3.1 Working mechanism

**FIGURE 1:**
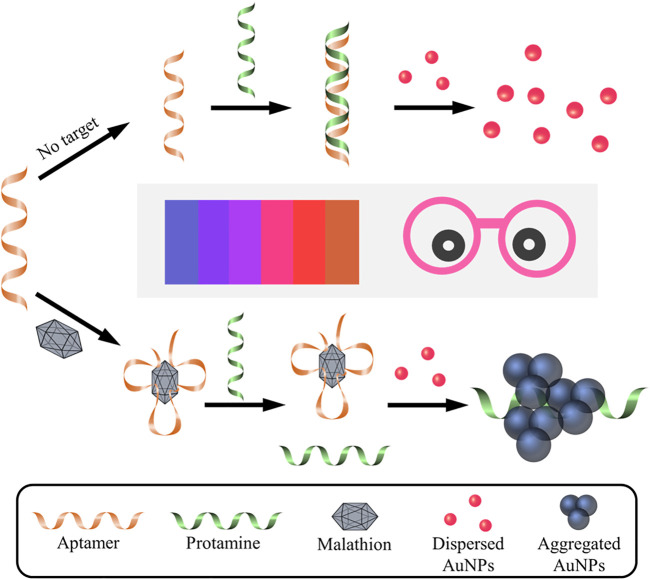
Schematic illustration of colorimetric aptasensor for malathion assay based on the utilization of protamine and AuNPs. Without the presence of malathion, aptamer probe is absorbed by protamine *via* electrostatic interactions, which makes it difficult for protamine to trigger the aggregation of AuNPs. As a result, the AuNPs remain well dispersed and exhibit red in color. On the contrary, the existence of malathion exhibits preferential binding affinity to aptamer, which is then unable to combine with protamine molecule. Consequently, protamine tends to mediate the aggregation of AuNPs and cause the color change to blue. It is noted that Pro, Apt, and Mal represent protamine, aptamer, and malathion, respectively. The color change of AuNPs solution can easily visualized by the naked eye and is closely related to the concentration of malathion in the solution.

The working mechanism of the designed aptasensor is depicted in Figure 1. In the absence of target malathion, the electrostatic absorption effect between the negatively charged aptamer probes and the polycation protamine molecules make it easy for them to form stable aptamer/protamine complex. The protamine molecules are then unable to interact with negatively charged AuNPs, which thus remain well dispersed in the solution with a red color. Nevertheless, the presence of malathion would consume the aptamer probes *via* the formation of malathion/aptamer complexes due to high affinity of aptamer towards malathion. The liberated polycation protamine would then be ready to induce the aggregation of AuNPs, imparting a color change from red to blue. There are several merits in the present sensing system for visual detection of malathion. Firstly, the sensing system is very simple, since there are no antibodies or enzymes involved. What are really demanded are only aptamer probes, protamine molecules, and unmodified AuNPs. Furthermore, the assay is easily performed with one mix-and-measure step, without the requirement of repeated washing and separation procedures. More importantly, the response signal can be readily observed by using the naked eye. Consequently, it is an enzyme- and device-free method for sensitive and rapid detection of malathion.

### 3.2 Characterization of AuNPs

AuNPs were synthesized by employing classic chemical methodology, in which trisodium citrate served a dual purpose, reduction agent and capping agent. AuNPs were then characterized by using UV-Vis spectrophotometer, visual detection, DLS, and TEM, as demonstrated in Supplementary Figure S1. The AuNP solution displayed red and had a strong absorption at around 520 nm (Supplementary Figure S1A). The hydrodynamic diameter of AuNPs was estimated to be 18.47 nm (Supplementary Figure S1B). As shown in Supplementary Figure S1C, the size of AuNPs determined by TEM was about 13 nm. Furthermore, AuNPs beard a negative surface charge and a Zeta potential of −37.31 mV was recorded (Supplementary Figure S1D), which was attributed to the coverage of citrate layer. All the results suggest that AuNPs with desired properties were fabricated and ready for further use.

### 3.3 Feasibility confirmation

**FIGURE 2:**
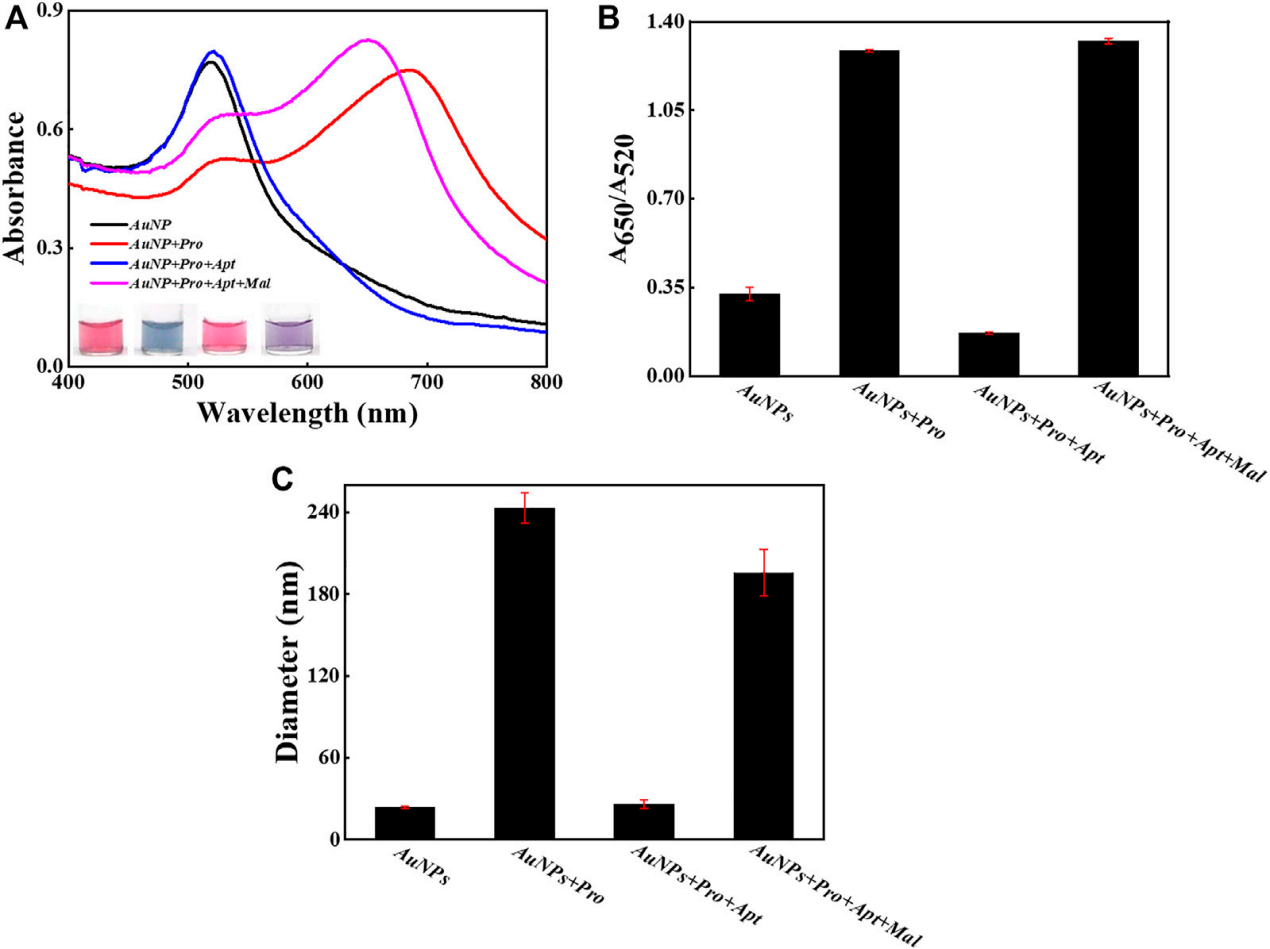
The feasibility examination. **(A)** The UV-Vis spectra of AuNPs and sample images with and without the introduction of malathion. **(B)** The values of A_650_/A_520_ in different samples. **(C)** The DLS results in different samples. The error bars represent the standard deviation of three independent measurements.

To verify the detection principle as demonstrated in Figure 1, several samples were intentionally prepared and their response signals were carefully compared and analyzed (Figure 2). As shown in Figure 2A, the absorption peak of bare AuNPs was around 520 nm, and it shifted to about 690 nm upon the introduction of protamine molecules, which suggests that AuNPs aggregated due to the electrostatic interactions between protamine and AuNPs. Interestingly, the absorption peak went back to about 520 nm after the addition of aptamer probes, which indicates that aptamer probes were able to reverse the aggregation behaviors of AuNPs. More importantly, the absorption peak moved to about 650 nm after the introduction of malathion, which confirmed the formation of AuNPs aggregation. The images of samples also revealed the status and color changes of AuNPs upon different stimulus. These visual detection results were consistent with those indicated by UV-vis spectra. The ratio of optical density between 650 nm and 520 nm is a typical indicator to illustrate the dispersion status of AuNPs. The results of A650/A520 (Figure 2B) support the fact that malathion was able to induce the aggregation of AuNPs. Moreover, the sizes of these samples were measured (Figure 2C). The AuNPs alone reported a hydrodynamic size of about 24 nm, and then grew to about 243 nm after mixing with protamine molecule. However, the hydrodynamic size returned back to around 26 nm upon the introduction of aptamer probes into the mixture containing AuNPs and protamine. The addition of malathion then permitted the AuNPs to aggregate with a hydrodynamic size of 196 nm. The results together verify that the working principle depicted in Figure 1 worked well as designed.

### 3.4 Condition optimization

**FIGURE 3:**
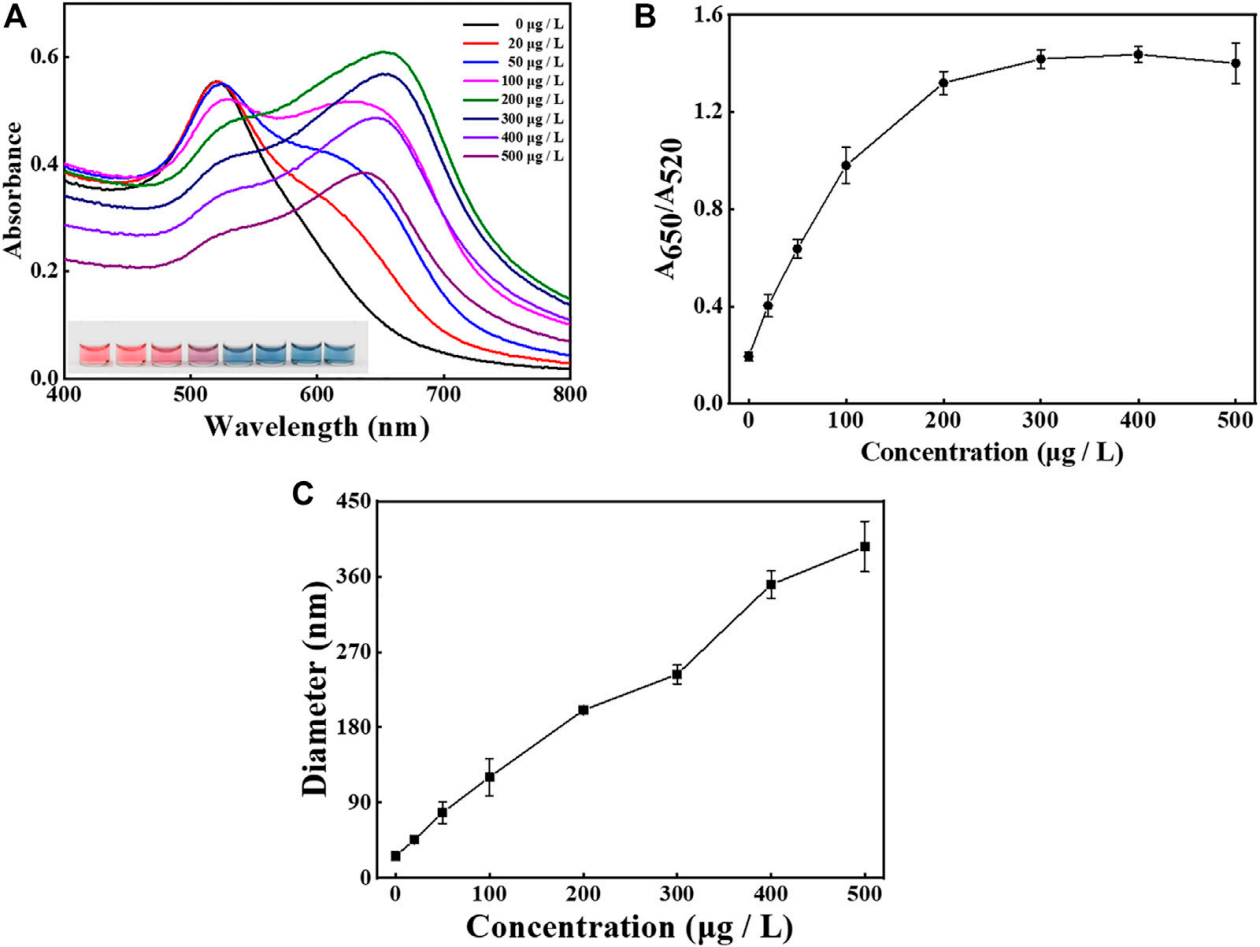
Condition investigation. The effect of protamine concentration (from 0 to 500 μg/L) on the sensing performance. **(A)** The UV-Vis spectra of AuNPs and sample images after the addition of protamine with varying concentrations. **(B)** The values of A_650_/A_520_ after the addition of protamine with varying concentrations. **(C)** The DLS sizes of AuNPs after the addition of protamine with varying concentrations. The error bars represent the standard deviation of three independent measurements.

**FIGURE 4:**
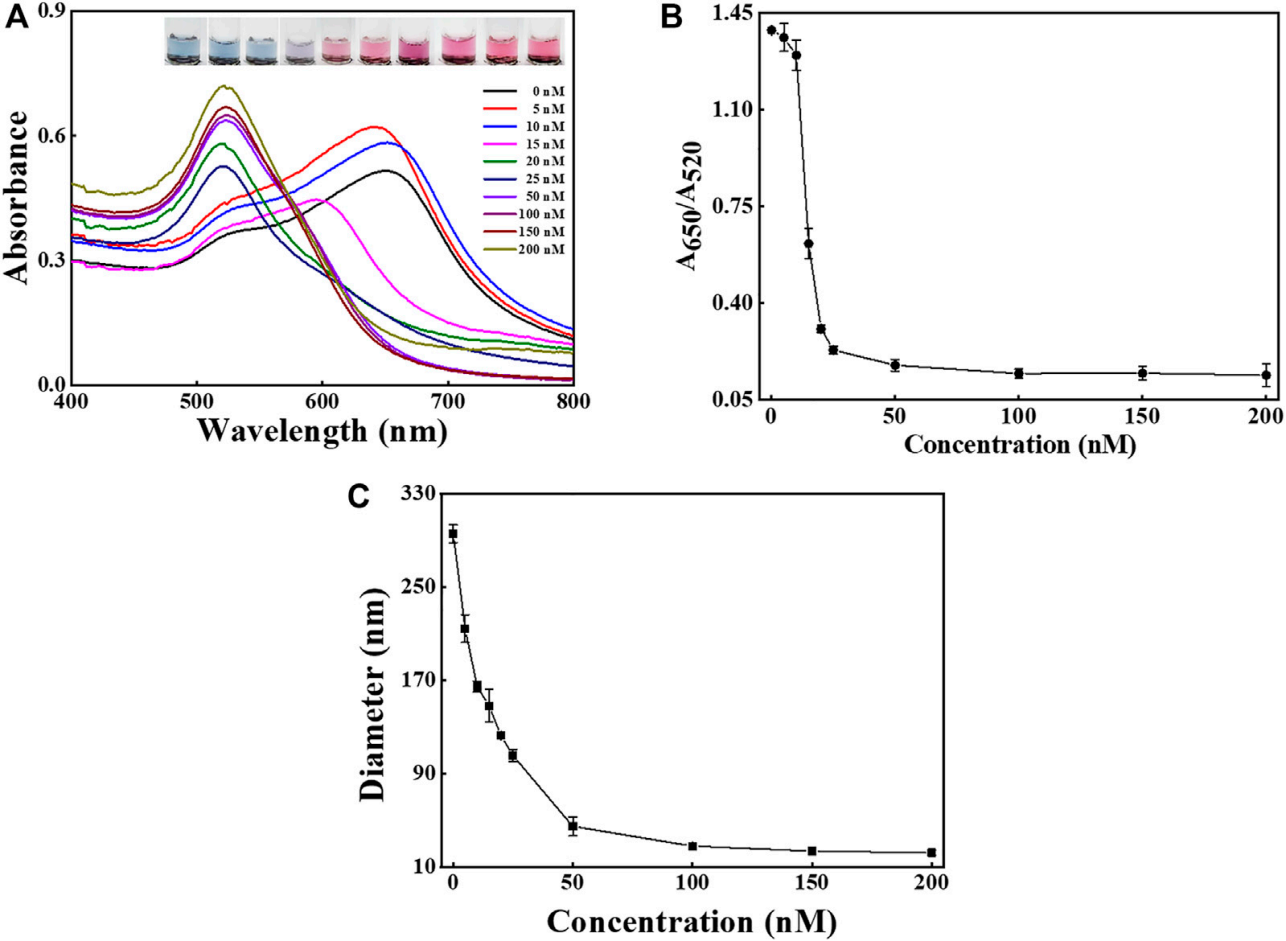
Condition investigation. The effect of aptamer amounts (from 0 to 200 nM) on the sensing performance. **(A)** The UV-Vis spectra and sample images of AuNPs after the addition of aptamer with different concentrations. **(B)** The values of A_650_/A_520_ after the addition of aptamer with different concentrations. **(C)** The DLS sizes of AuNPs after the addition of aptamer with different concentrations. The error bars represent the standard deviation of three independent measurements.

To achieve the optimal performance, several important experimental parameters in the sensing system were optimized, including the concentrations of protamine (Figure 3) and aptamer probes (Figure 4), as well as the incubation time between the aptamer and malathion (Supplementary Figure S2). To study the effect of protamine concentration, AuNPs were mixed with varied protamine concentrations ranging from 0 to 500 μg/L. As shown in Figure 3, the UV-Vis spectra, actual images, and DLS measurements were recorded and plotted for further analyses. The UV-Vis spectra shifted red with the growing of protamine concentration (Figure 3A). Meanwhile, the color of samples changed from pink to blue as the protamine increased, and then remained unchanged after the concentration reached 300 μg/L. As presented in Figure 3B, the value of A_650_/A_520_ gradually rose and then stabilized when the protamine concentration arrived at 300 μg/L. Consequently, 300 μg/L was chosen for subsequent experiments. It is also observed from Figure 3C that the sizes of AuNPs had a linear dependence on the concentrations of protamine molecules.

The effect of aptamer amounts on the dispersion of AuNPs and the performance of the method was also examined, as shown in Figure 4. The UV-Vis spectra shifted blue as the concentration of aptamer increased (Figure 4A), and the images reveal that the solution color varied from blue to pink. As presented in Figure 4B the ratio of A_650_/A_520_ gradually decreased and became stable at aptamer concentration higher than 150 nM. The result indicates that 150 nM was sufficiently high to protect the stability of AuNPs. The DLS results suggest that the hydrodynamic diameter of AuNPs sharply dropped when the aptamer amounts rose and then levelled off at about 150 nM (Figure 4C).

In order to keep a good balance between the effective binding and rapid detection, the incubation time between malathion and aptamer probes was also investigated, as shown in Supplementary Figure S2. The UV-Vis spectra of several time points kept unchanged (Supplementary Figure S2A) and the A_650_/A_520_ was stable (Supplementary Figure S2B). The results demonstrate that the interaction between malathion and the corresponding aptamer might be completed within 10 min. As a result, 10 min was chosen for the subsequent experiments.

### 3.5 Sensitivity and specificity evaluation

**FIGURE 5:**
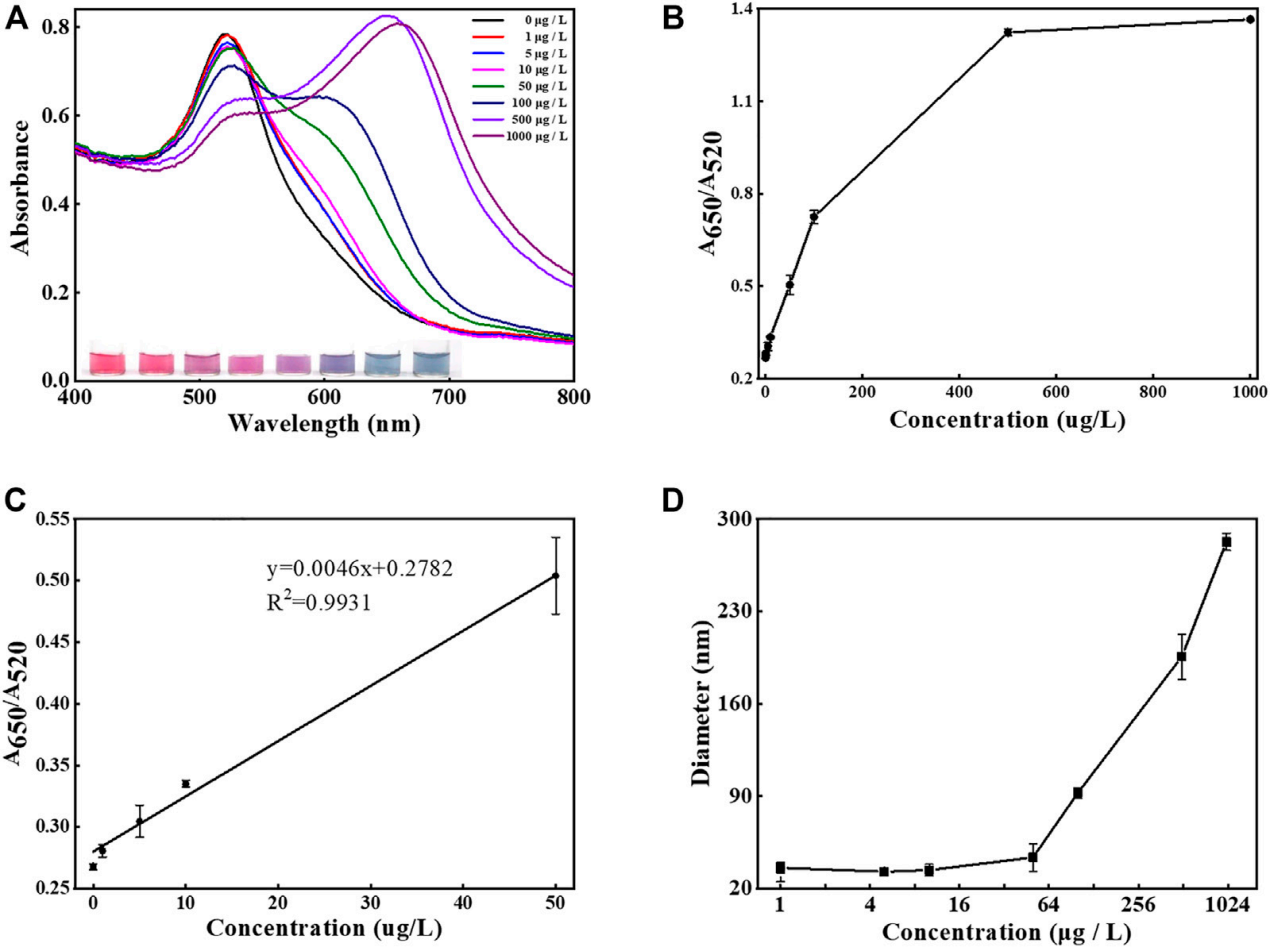
Sensitivity determination. **(A)** The UV-Vis spectra and images recorded after the introduction of malathion with concentrations from 0 to 1,000 μg/L. **(B)** The dependence of the values of A_650_/A_520_ against various target concentrations from 0 to 1,000 μg/L. **(C)** The linear relationship between the values of A_650_/A_520_ and the concentration of malathion from 0 to 50 μg/L. **(D)** The dependence of the DLS sizes of AuNPs in solution against various target concentrations from 0 to 1,000 μg/L. The error bars represent the standard deviation of three independent measurements.

After careful evaluation of optimized reaction conditions, the sensing performance of the aptasensor was assessed by utilizing UV-vis spectroscopy, visual observation, and DLS. To study the sensitivity of the designed method, the malathion with concentration ranging from 1 to 1,000 μg/L was added followed by the signal recording under the optimized experimental conditions (Figure 5). As seen in Figure 5A, when the concentrations of malathion grew higher, the absorption spectrums decreased at 520 nm and meanwhile increased at 650 nm. The color of samples also changed from red to blue. The dependency of A_650_/A_520_ with the malathion levels was plotted in Figure 5B. A_650_/A_520_ increased linearly with the concentrations of malathion varying from 1 to 50 μg/L, as shown in Figure 5C. The limit of detection (LOD) was theoretically calculated as 1.48 μg/L with a regression coefficient *R* ^2^ = 0.9931 based on the 3σ/s rule, in which σ is the standard deviation of the background signal and s stands for the slope of the calibration plot. It is noted that the LOD is below the maximum residue limit of malathion (10 μg/L) set by China’s Agriculture Ministry (GB 2736-2021). The performance of the current assay method was compared with previously reported approaches (Table 1). It is noted that the detection limit of the current method is poorer than some other strategies, such as fluorescent (Chen et al., 2020), electrochemical (He et al., 2018), and chemiluminescent sensors (Wu et al., 2021). Fortunately, it is still sufficiently sensitive to meet the application requirement for malathion monitoring in drinking water. As reported in previous research, GNP-based DLS methods are able to achieve significantly enhanced sensitivities for the detection of DNA (Dai et al., 2008), proteins (Jans et al., 2009), and As(III) ions (Kalluri et al., 2009). But unlike the unmodified GNPs used in this work, the surfaces of GNPs need to be further functionalized to realize the assay.

**TABLE 1:** The performance comparison of malathion detection methods.

Methodology	Linear range (μg/L)	LOD (μg/L)	Ref
electrochemical	—	6.7	Wang et al. (2016)
	1-10000	0.16	He et al. (2018)
fluorescence	100-25000	10	Wang et al. (2019)
	3.3-330	0.47	Chen et al. (2020)
surface-enhanced Raman scattering	167-3333	167	Nie et al. (2018)
	—	123	Albuquerque et al. (2015)
resonance Rayleigh scattering	12-800	1.7	Huang et al. (2019)
chemiluminescence	—	1.57 × 10^−4^	Wu et al. (2021)
localized surface plasmon resonance	—	1.8×10^3^	Dissanayake et al. (2019)
colorimetric	10-120	3.1	Xu et al. (2021a)
	16.6-233	3.93	Li et al. (2019)
	8.3-5333	7.5	Liu et al. (2021)
	1-5000	14	Faghiri et al. (2021)
	0-50	1.48	This work

**FIGURE 6:**
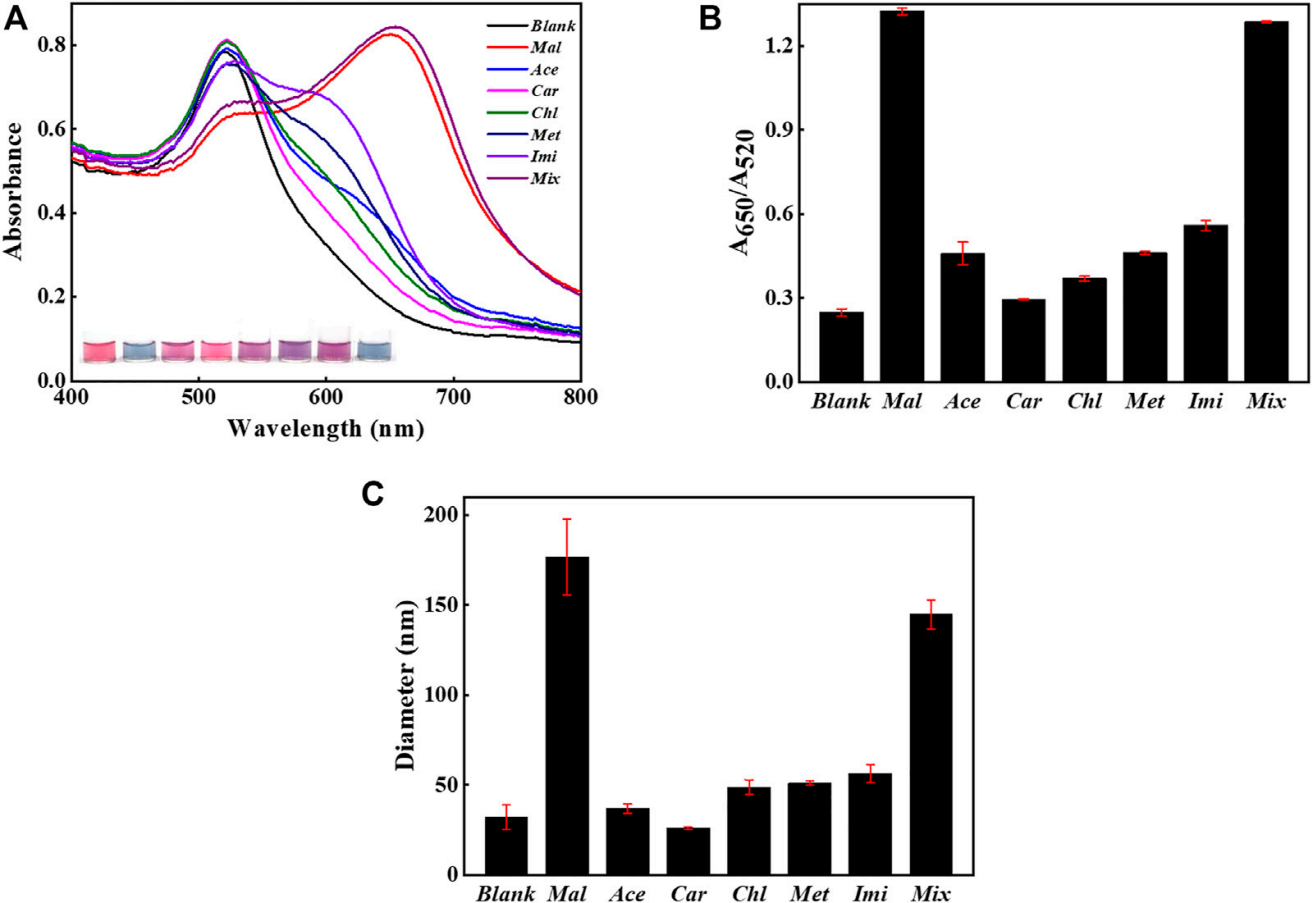
Selectivity evaluation. The sensing method when challenged with 1,000 μg/L non-specific substances alone or coexisting with the presence of 500 μg/L malathion. **(A)** The UV-Vis spectra of AuNPs and sample images after the introduction of non-specific analytes with or without the presence of malathion. **(B)** The values of A_650_/A_520_ in different samples. **(C)** The DLS sizes of AuNPs in different samples. The error bars represent the standard deviation of three independent measurements.

The specificity was accessed by challenging the system with potential interfering agents such as acetamiprid, carbaryl, chlorpyrifos, methamidophos, and imidacloprid, as displayed in Figure 6. The presence of malathion alone or co-existing with interfering agents caused a significant change in the UV-Vis spectra and solution color (Figure 6A). But the sensing system did not have response upon the addition of non-specific targets, since the UV-Vis spectra and solution color remained unchanged compared with the blank sample. Additionally, the value of A_650_/A_520_ clearly distinguished the signal produced by malathion from those caused by the other competitive organophosphorus pesticides, as found from Figure 6B. The co-existence of non-specific pesticides did not affect the sensing of malathion. As demonstrated in Figure 6C, the samples containing malathion also reported considerably higher sizes than those involving un-specific analytes. All these results confirm that the constructed sensing strategy was highly specific for malathion due to the high affinity of aptamer to malathion. Moreover, the sensing system was highly stable even in the presence of a great number of interfering agents, which guarantees the detection reliability.

#### 3.6 Practical application

In order to assess the capability of the detection method for real sample, the malathion in tap water was also detected and analyzed. Malathion with various concentration (10 μg/L and 50 μg/L) were individually spiked into tap water, The results were given in Table 2. It is shown that the acceptable recovery rates ranging from 98.9% to 109.4% were achieved. The data indicates that the proposed aptasensor had a promising potential in real applications.

**TABLE 2:** The recovery tests of malathion spiked in tap water using the current method.

Sample	Target added (μg/L)	Target found (μg/L)	Recovery (%)
1	10	9.9 ± 3.3	98.9
2	50	54.7 ± 5.9	109.4

To confirm the detection accuracy, HPLC method was used for the built of calibration curve (Supplementary Figure S3A) and the recovery rates of several concentrations of malathion spiked into tape water were determined, and the results could be found from Supplementary Figure S3b; Table 3. The data reveals that the present method was accurate for malathion assay in real sample and held a promising for further applications.

**TABLE 3:** The recovery tests of malathion spiked in tap water using HPLC.

Sample	Malathion added (μg/L)	Malathion detected (μg/L)	Recovery rate (%)
1	150	150.3 ± 15.3	100.2
2	300	320.4 ± 19.5	106.8
3	600	691.3 ± 45.7	115.2

There are some limitations in the current work. First of all, although barely GNPs are easily synthesized and serve as excellent color indicators, their colloidal stability is highly sensitive to the choice of buffer, salt concentration, and pH values. To improve their stability and avoid undesired aggregation, surface functionalization may provide an effective solution to this issue. Moreover, the length of aptamer probes is also an important factor that may influence the stability and eventually the sensing performance for malathion assay. Therefore, the choice of aptamer strands and the reaction buffer is an essential step in the construction of this kind of colorimetric sensors.

## 4 Conclusion

Aptamers, a superior alternative to antibodies, have emerged as powerful bio-inspired receptors for the design and applications of new biosensors, especially combining with nanomaterials. Inspired by target recognition in nature, an aptamer-mediated, gold nanoparticle-based sensing approach was deployed to quantify malathion in a robust and selective fashion. The assay of malathion could be conducted within 40 min in a add-and-observe manner by using the naked eye. This label-, antibody-, and device-free method was able to detect malathion as low as 1.48 μg/L (0-50 μg/L) and held a promising potential for monitoring malathion in drinking water. The detection accuracy was also verified by the HPLC method. By selecting proper aptamer probes for relevant targets of interest, the sensing concept in this work can be easily extended for the detection of other analytes. This will be our future concern. The construction of powerful aptasensor may open a new path for efficient reporting of pesticides, which benefits the development of agriculture, environmental protection, food control, and public health.

## Data Availability

The original contributions presented in the study are included in the article/Supplementary Material, further inquiries can be directed to the corresponding author.
