# Supplementary material for: Bio-inspired aptamers decorated gold nanoparticles enable visualized detection of malathion
Source: Front Bioeng Biotechnol. 2023 Mar 3;11:1165724. doi: 10.3389/fbioe.2023.1165724 (PMC10020530; doi:10.3389/fbioe.2023.1165724)
Supplement: Supplementary file 1 [file DataSheet1.docx]

**Bioinspired Aptamers Decorated Gold Nanoparticles Enable Visualized Detection of Malathion**

Peng Li,^1,2^*^✝^* Haonan Zhan,^1^*^✝^* Sijian Tao,^1^*^✝^* Zhuohao Xie,^1,2^ and Jiahao Huang^1,2*^

^1^*School of Biomedical Engineering, Southern Medical University, Guangzhou 510515, P. R. China*

^2^*Department of Critical Care Medicine, Affiliated Hospital of Guangdong Medical University, Zhanjiang 524000, P. R. China*

** Corresponding Author. Email:* [*jhuangaf@connect.ust.hk*](mailto:jhuangaf@connect.ust.hk) *(Jiahao Huang)*

*^✝^ These authors contributed equally to this work.*

**Figure S1** The characterization of AuNPs. (a) The UV-Vis spectra of AuNPs and sample images. (b) The dynamic light scattering (DLS) measurement. (c) The transmission electron microscope (TEM) observation. (d) The Zeta potential recording.

**Figure S2** Condition investigation. The effect of incubation time (from 10 min to 60 min) between malathion and aptamer on the sensing performance. (a) The UV-Vis spectra recorded after different incubation time. (b) The values of A_650_/A_520_ after different incubation time.

**Figure S3** HPLC results. (a) The calibration curve of HPLC method between the peak area and malathion concentrations varying from 100 to 1500 μg/L. (b) The response of several selected concentrations of malathion spiked into tape water detected by HPLC method.
